# Record of Meteorological Conditions at Buffalo, for March, 1856

**Published:** 1856-05

**Authors:** Sanford B. Hunt


					﻿ART. VI.—Record of Meteorological Conditions at Buffalo, for March,
1856. By Sanford B. Hunt, M. D.
| |	—————_	,___________
i
3	=
«	. tA	£	.	g
3	2	2	-•
a	'	—	.V
PS	S IS 55	-! ‘s	a	-J .22
SP	. h	a	-c“
*	'S it
= « .S .S	2	t? g
“ Es	£	£	"	"	t>'	&	it 10
a o	o	o	S	a	c	o	o a
•- a	c	a	o	-	e	c	—<
d X	®	®	O	X	X	X	00
•ajnjv.iodcuaj, I	\
jo oiRnn jsojBoigl	m o d ao	tJJ	-rf	x	co	t—	co co	co	no co	co	m in m	t--	cs	t- o	co
•	aoijB.dB.ig jol	co co co co	co	co	co	co	co	co	co	co	co.	co	co.	co .	I —
q atuaj, u«aj\[	iriooiricri	—2	d	d’	co	—f’cd	oc	od—	d’—	in ci o	b4	ci	ci ci	cd
2	_____________d — d — —i |________dd d_____________d d CO CO cococod d d 'd
p> . ajnJB CO CO CO CO CO .CO CO CO CO CO	CO COCO	CO CO CO CO .t'WM j
-jaatuaj. ubojv] co o’ t~ cd ci -d co cd in in «-4	—I o ci co cd ci ci o o cd -d !
________________<?> C7 CQ CQ	—<	|	O) TM 'M ''N	C*? CO CO CO CO CO CO CO O1 CO O> O '(71
•a01jB.dB.13	...	!
2 jo ’dujax co' co' cd -4 cd t-4 cd ci cd rd cd cd ci co cd dd 4 d ad ci oi d
C	d — — d d I —•	— d d	co d d CO d CO CO CO d — d — d
j* . £	I	7
£ < i'i 0Jn}‘	16 o to 1.0 Tf U ^4 ci ai 0 ci to o ci to co co 0 co
5	. 72_____fr> ~' frt.fr* fr* I —1	(NO?	__COCQCOCOG^ CO CO CO (7!	—‘ CM CM (71
I	q a> j |	L<*	t*" £*■ L-*- ►>.	£>-
a '	o§g°£ K*MX'	X XXXX	«2	X	fi X
2 * .........................................
c_________________'co -r3<	d o co	d — d d O CO O	d	O	O	PC d	O	O	O	o	o co	two
spnoo jo j.ui y	|O x	o o o	ooooooooo	o	o co	»	g>	o o	o	o o	o o	o	|
I 0 -aopB.dBAg...................... .	.	.............| in
£	10	-data [,	d’ fd	co’ o’ rd	co’ <--' d’ -c' ci -st d' -cP	—'	ci -*	cd	cd —	cd	—	cd	cd oo'	ci	cd ci	-dcd	co	ico
co d	d co d	d d —< — d co	co	co co	co	co co	or	co co_co	co co	d d d	d	1 ot
•	‘"I	CO
3	i? 8Jllt<Itu®xLt- <—•1/5- 7j i^'ad ao' cd aS rd cd to' cd id cd cd — ci co' -4 id o' o' ci id d' >d jndo ltd
—	• CO (71 *71 20 O') O1 0^ —•	—• — OlCOCOCOCOCO'O'CO'^’^T’^O'O1'^ co co co (71 (71 (7? co co
"*1	“ -d | .iSESjS	p: ^'iS p4 b pi
N C§go2 iM-S X X XP^ X X X X XX’ x’ X CO x’ x’x'x’x X X p4 X ki &2 X <5
Q r- .	........................... ...............................
____________X1 -r cc r .O' io d O' ic,	01 r, o d -f - c 01 - - to " co co d
spni)[3jl> J.UIVI® '—iC C	3 C	C 3 O CC Q is US O Kt O •'. 10 O'3 X C C C O C lO cc |
® -aoijB,dBA3 ....	. <d-d . .	.................................
o jo -daia l •-■ 1-- - X x ido d - - d m d d is c> -r o. c - 'C 'c o	x ,< cj o c no
p ’	d d d d d 1 1	—dd — —'dddddcocodd — —■ — d^
J _: o J	|	.	p,
. >.	_• _•	r—- ® 2 o cd ci t—’ cd -d ci ci —‘ id t-- <4 cd id id 0 id <d cd rd <d ci
<!	33 i	IdddCO d H I I—I— dd—'dCOddddCOCO CO d C? r^ rl d d
-d	. iS pi	,^'jS	.P?
< r° § o °S 24 02 o; 02 X 02 02 X X 02 02 02	02’ 02’	02 02	X X	X JZj
s? — o o " cc"i -r d cc cc ?) -2dcoooco — ooo^<r-(ddcocn. co—______
«pnoo jo j, m y | a> x o co so o o o o o 0’0 o 00 00 m §> g> woooooooo|
/. -area jo-nj
a	|t~dcit~cncooo —	ddoo —co	d d	-t	co	co co	.co	.	. co	o	d —	m	d
•uiOJBg jo 'ujl^ -2 m o cd	cd cd cd	= cd ci o' o'	o'	o' o' o'	o' o	cd	cd oi	o co	o	o ci	co	cd o	o	o'
(71 v? /v? Tt	ci cm co	cq co co	co	cq co cc	co co	ci	ci (71	CO <71	rc	7? V?	(71	cc cc	co_	co
.OTBfT|-. d co	id"^xFcdo' - d'co	x d	o	- d	co	g:	0	x	d'o|	,
				

